# Antimicrobial prophylaxis and post-chemotherapy neutropenic fever in patients with leukemia: comparisons of C-reactive protein, procalcitonin and immediate fever outcome measures between those with and without prophylaxis, and the implications for practice

**DOI:** 10.1007/s00520-021-06325-3

**Published:** 2021-06-08

**Authors:** Choi Wan Chan, Alex Molassiotis, Harold K. K. Lee

**Affiliations:** 1 School of Nursing, The Hong Kong Polytechnic University, Hung Hom, Kowloon, Hong Kong; 2 Department of Medicine and Geriatrics, Princess Margaret Hospital, Kowloon, Hong Kong

**Keywords:** Leukemia, Post-chemotherapy neutropenic fever, Antibiotic or antifungal prophylaxis, C-reactive protein, Procalcitonin, Risk–benefit of prophylaxis

## Abstract

**Purpose:**

The efficacy of prophylactic antimicrobial treatment renders challenges in patients with leukemias receiving chemotherapy. The study aimed to compare differences in C-reactive protein (CRP) and procalcitonin (PCT) at presentation and the immediate outcome measures of post-chemotherapy NF between patients with and without antimicrobial prophylaxis.

**Methods:**

A 5-year observational study included 282 NF episodes in 133 leukemia patients requiring hospital care from January 2014 to May 2019. We collected demographic characteristics, laboratory data of blood cell counts and inflammatory biomarkers, and immediate outcome measures of NF, including microbiologically diagnosed infections, presence of predominant pathogens, required modification of antibiotics during NF, adverse medical complications, total fever duration, and deaths. We evaluated data between patients with and without prophylaxis.

**Results:**

Of patients, 77.3%, 68.4%, and 20.6% had antibiotic prophylaxis, antifungal prophylaxis, and no prophylaxis, respectively. There were totally 15 deaths—13 with antibiotic prophylaxis and 10 with antifungal prophylaxis. CRP, PCT, and immediate outcome measures of NF did not show significant differences between those with and without antimicrobial prophylaxis. Although between-group differences showed no statistical significance, higher median fever duration, CRP and PTC values, and higher proportions of NF requiring modification of antibiotics were found more frequently in those with antimicrobial prophylaxis than in those without.

**Conclusion:**

The benefits of using antimicrobial prophylaxis were less supported. Enhancing diagnostic laboratory and medical complication surveillance and periodic evaluation of institutional data during post-chemotherapy neutropenia and NF in relation to antimicrobial prophylaxis is promising in providing insights to redefine the risk–benefit accounts of using prophylaxis.

## Introduction

Post-chemotherapy immunocompromised hematological patients are at risk of bacterial and fungal infections. Leukemias constitute a large proportion of hematological malignancies. It is common for neutropenic patients with leukemias to receive antimicrobial prophylactic treatments in post-chemotherapy. Antibiotic prophylaxis for high-risk patients with prolonged duration of neutropenia has been recommended in international guidelines [1–5]. A systematic review showed that antibiotic prophylaxis in post-chemotherapy afebrile neutropenic patients significantly reduced the occurrence of fever, indicators of infection, microbiologically documented infections, and infection-related and all-cause mortality [6].

In a randomized study, patients with hematological malignancies who developed neutropenic fever while on antibiotic prophylaxis had a lower probability of response to first-line empirical antibiotic treatments and a delay in fever resolution compared with those randomized to no prophylaxis, and there was no reduction in hospital stays and cost among those receiving prophylaxis when compared to those without [7]. There is also considerable literature showing that the emergence of antimicrobial resistance and the spread of multidrug-resistant pathogens are associated with the use of antibiotic prophylaxis [8–11]. Putting the extensive use of prophylaxis in the context of increasing antibiotic resistance, Australian guidelines advise against the routine use of prophylaxis for neutropenia [12]. In view of the pharmacovigilance reports, the U.S. Food and Drug Administration has attended to the issue of antibiotic toxicity and released warnings against unnecessary use of antibiotics due to their association with disabling and potentially permanent side effects that involve the central nervous system, nerves, peripheral neuropathy, muscles, joints, tendinitis, tendon rupture, confusion, and hallucinations [13]. Investigators have even drawn attention to the fact that the potential benefit of prophylaxis in lowering the rate of infection was demonstrated in regions with low to moderate antibiotic resistance rates, suggesting that it might not be applicable in regions with a high prevalence of resistant pathogens [14–16]. Therefore, a clear-cut benefit regarding the efficacy of antibiotic prophylaxis appears less assured.

*Candida* spp. and *Aspergillus* spp. account for most of the fungal infections occurring during neutropenia in patients with hematological malignancy (HM) [17]. Although antifungal prophylaxis is used against *Candida* spp., *Aspergillus* has surpassed *Candida* as a cause of invasive fungal infections [18]. Antifungal therapy reducing the diagnostic sensitivity of a galactomannan enzyme immunoassay for fungal infection has also been reported [19]. As such, these offer challenges in prophylactic antifungal therapy, confirming that the use of prophylaxis might have an impact on the choice of strategy in the management of post-chemotherapy neutropenia [20].

Local policies on the use of antimicrobial prophylaxis are often in line with international antimicrobial stewardship programs. It is essential that specialists and clinicians examine the impact of antimicrobial prophylaxis that is apparently suggested [21]. Institutional research data analyses and clinical investigations of antimicrobial prophylaxis in post-chemotherapy NF are useful to inform policies and practices, but these appear rarely. The purpose of the present study was to examine the impact of antimicrobial prophylaxis in post-chemotherapy NF in leukemias by comparing differences in inflammatory biomarkers of C-reactive protein (CRP) and procalcitonin (PCT) at the onset of NF and immediate outcome measures of NF between those with and without prophylaxis. CRP and PTC have been widely studied as inflammatory biomarkers related to NF [22–24].

## Methods

### Sample, design, and definition of an NF episode

The study was part of a larger university and institutional review board-approved observational study investigating clinical profiles and patient-reported symptoms and their relationships with inflammatory biomarkers and clinical prognostic data in post-chemotherapy NF patients with hematological malignancies. The present study included data from adult patients with leukemias (acute/chronic myeloid leukemia (AML and CML) and lymphocytic leukemia (ALL and CLL)), admitted between January 2014 and May 2019 requiring clinical care for post-chemotherapy NF in the hematological units of a regional acute hospital. Febrile episodes were identified retrospectively and prospectively from January 2014 to December 2016 and June 2017 to May 2019, respectively. Informed consent was obtained from participants for the prospective collection of data. We used the same study pro forma to collect and retrieve data from the patient medical records for both groups. The inclusion criteria consisted of patients ≥ 18 years, diagnosed with leukemias and presenting with an episode of NF. Fever and neutropenia were defined as a temperature ≥ 38.3 °C, or ≥ 38 °C for two episodes more than 1 h apart, and an absolute neutrophil count (ANC) < 0.5 × 10^9^ cells/L, or < 1 × 10^9^ cells/L, expected to decrease below 0.5 × 10^9^ cells/L within 48 h [25]. We defined an NF episode as the duration from the onset of NF to the point of NF subsiding (i.e., < 37.5 °C), provided that the temperature to which it subsided (i.e., < 37.5 °C) was persistent for 48 h (a time point of 48-h afebrile). An afebrile temperature was defined as a temperature ≤ 37.4 °C. Subsequent episodes of fever in the same neutropenic patient were included and counted as separate, independent NF events.

### Study measures and data collection

Antimicrobial prophylaxis referred to any use of antibiotic and antifungal therapy within the 7 days prior to NF presentation. Blood cell counts, ANC, and inflammatory biomarkers of CRP and PCT at presentation, as indicated in patients’ medical records, were collected. The outcome measures of fever as documented in the medical charts included data collection of microbiologically diagnosed bacterial and fungal infections (MDBIs and MDFIs), presence of predominant pathogens, whether modification of antibiotics was required during NF, medical complications in the first 3 and 5 days of an NF episode, total fever duration, and death. MDBIs and MDFIs were defined as infectious bacterial and fungal pathogen(s), respectively, detected in laboratory cultures. Medical complications included hypotension (systolic arterial pressure < 90 mmHg); arrhythmia; ICU admission due to septic shock; respiratory insufficiency, defined as oximetry saturation < 95% requiring oxygen therapy; documented altered mental status and acute kidney injury; and infiltrates on a chest radiograph [26].

### Statistical analysis

To describe the sample demographics, clinical laboratory and microbiological data, and NF clinical outcome parameters, we used proportions, mean (M) and standard deviation (SD), or median (Med) and range, as appropriate. In categorizing data, the normal values for PCT and CRP were taken as < 0.5 ng/mL and ≤ 5 mg/L (i.e., ≤ 5 μg/mL), respectively [27]. Proportions for the categorical variables were compared by Chi-squared test. Continuous data between the groups were compared by Mann–Whitney U test. A *p* < 0.05 level of significance was used. Each NF episode was taken as an individual event in the analysis of data.

## Results

### Patient demographic and clinical characteristics

**Fig. 1:**
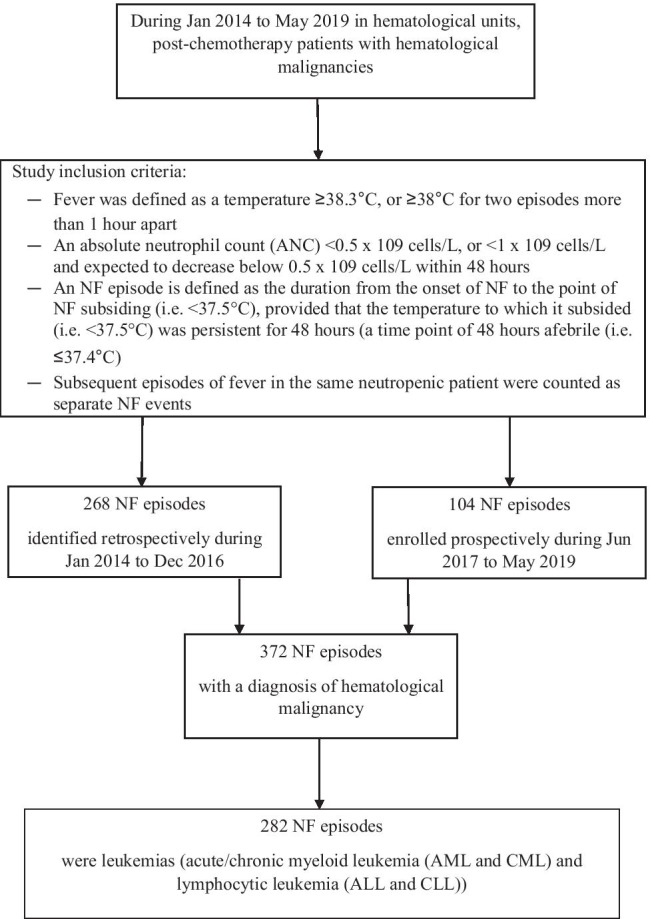
Flow chart

In the 5-year observational study, data included 282 NF episodes from 133 patients with leukemias (Fig. 1). Table 1 summarizes the demographic and clinical characteristics of the sample. The mean age of the sample was 51.1 (SD = 12.1), with 54.1% being male. AML was the most common underlying hematological malignancy, accounting for 76.2% (*n* = 215) of NF episodes. In terms of antimicrobial prophylaxis prior to the onset of NF, 77.3% (218 episodes) and 68.4% (193 episodes) of cases had used antibiotic and antifungal prophylaxis, respectively. Over 66% (187 episodes) had used both antibiotic and antifungal prophylaxis, and 20.6% (58 episodes) had not undergone prophylactic antimicrobial therapy. Over two-thirds (67%, 189 episodes) of the sample (*n* = 282) required antibiotic modifications after the first-line empirical antibiotic had been administered.

**Table 1:** Sample demographic and clinical characteristics (*n* = 282 NF episodes)

Variables	Frequency (%)
Gender	
Male	72 out of 133 (54.1)
Female	61 out of 133 (45.9)
Hematological disorders	
Acute myeloid leukemia (AML)	215 (76.2)
Acute lymphocytic leukemia (ALL)	52 (18.4)
Chronic myeloid leukemia (CML)	6 (2.1)
Chronic lymphocytic leukemia (CLL)	9 (3.2)
Microbiologically diagnosed bacterial infections (MDBIs)	62 (22.0)
Microbiologically diagnosed fungal infections (MDFIs)	53 (18.8)
Pathogens	
Gram-negative bacterial pathogens	36 (12.8)
Gram-positive bacterial pathogens	32 (11.3)
*Escherichia coli* (*E. coli*)	20 (7.1)
Methicillin-resistant *Staphylococcus aureus* (MRSA)	17 (6.0)
Prophylaxis before the onset of NF	
Antibiotic	218 (77.3)
Antifungal	193 (68.4)
Antibiotic and antifungal	187 (66.3)
No prophylaxis	58 (20.6)
First-line empirical antibiotic at the onset of NF	
Sulperazon (cefoperazone-sulbactam)	137 (48.6)
Tazocin (piperacillin-tazobactam)	90 (31.9)
With modification of antibiotic during FN	189 (67.0)
Serious complications in the first 3 and 5 days of NF, during NF	
Documented chest X-ray consolidation/infiltration	40 (14.2), 53 (18.8), 62 (22.0)
Hypotension	36 (12.8), 41 (14.5), 56 (19.9)
Impaired respiratory function	33 (11.7), 37 (13.1), 57 (20.2)
Severe bleeding requiring transfusion	12 (4.3), 12 (4.3), 18 (6.4)
Arrhythmia	9 (3.2), 11 (3.9), 15 (5.3)
Documented confusion/altered mental state	7 (2.5), 10 (3.5), 23 (8.2)
Required admission to intensive care unit	5 (1.8), 7 (2.5), 9 (3.2)
Heart failure	4 (1.4), 5 (1.8), 7 (2.5)
Disseminated intravascular coagulation	3 (1.1), 3 (1.1), 3 (1.1)
Renal failure	3 (1.1), 3 (1.1), 5 (1.8)
Deaths	15 out of 133 patients (11.3)
AML	14
CLL	1
MDFIs	6
MDBIs	5
Gram-negative bacterial pathogens isolated	2
Gram-positive bacterial pathogens isolated	2
Gram-negative and gram-positive bacterial pathogens isolated	1
With antibiotic prophylaxis	13
With antifungal prophylaxis	10
Variable (range)	Mean (SD), median
Age (20–83 years)	57.1 (12.1), 58
NF duration (3–993 h)	180.0 (178.1), 124
ANC at the onset of NF (0–0.9 × 10^9^ cells/L)	0.18 (0.28), 0.0
Hemoglobin level at the onset of NF (1.7–11.8 g/dL) (*n* = 218)	7.8 (1.5), 7.8
Platelet count at the onset of NF (2–221 × 10^9^ cells/L) (*n* = 212)	28.8 (37.7), 15
Albumin level at the onset of NF (13–96 g/L) (*n* = 200)	34.7 (7.0), 35
Creatinine level at the onset of NF (27–4725 µmol/L) (*n* = 205)	91.1 (326.0), 66
Bilirubin level at the onset of NF (4–447 µmol/L) (*n* = 198)	19.6 (32.2), 15
CRP at the onset of NF (0.9–270 mg/L) (*n* = 125)	76.8 (56.9), 64.0
*CRP > 5 mg/L at the onset of NF (6–270 mg/L) (*n* = 124)	77.4 (26.7), 64.5
PCT at the onset of NF (0.0499–318 ng/mL) (*n* = 182)	2.89 (24.0), 0.18
**PCT ≥ 0.5 ng/mL at the onset of NF (0.54–318 ng/mL) (*n* = 35)	14.3 (53.8), 1.20

*NF* neutropenic fever; *n* sample size; *SD* standard deviation; *ANC* absolute neutrophil count; *CRP* C-reactive protein; *PCT* procalcitonin; *abnormal values of CRP, when CRP normal reference value is ≤ 5 mg/L; **abnormal values of PCT, when PCT normal reference value is < 0.5 ng/mL

Among the parameters with abnormal ranges, the median values of CRP and PCT were 64.5 mg/L and 1.2 ng/mL, respectively. The median duration of the NF was 124 h (5.2 days). There were 22% (62 episodes) of MDBIs and 18.8% (53 episodes) of MDFIs. Gram-negative pathogens were slightly predominantly isolated (12.8%, 36 episodes) when compared with gram-positive pathogens (11.3%, 32 episodes). The most common pathogens isolated were *Escherichia coli* (*E. coli*) (7.1%, 20 episodes) and methicillin-resistant *Staphylococcus aureus* (MRSA) (6.0%, 17 episodes). In this sample (*n* = 133), totally 15 patients died (11.3%). There were 13 deaths among those who had used antibiotic prophylaxis and 10 among those who had used antifungal prophylaxis. Documented abnormal chest X-ray (infiltration/consolidation), hypotension, and impaired respiratory function were the major adverse medical complications of NF.

### Comparison of differences in outcome measures of fever and inflammatory biomarkers of CRP and PCT between those with and without antimicrobial prophylaxis

Table 2 shows no statistically significant differences between MDBIs/MDFIs in those with and without antibiotic/antifungal prophylaxis, respectively. The presence of gram-negative and gram-positive pathogens was not significantly different between groups with and without prophylactic antibiotic treatment. There were no statistically significant differences for required antibiotic modification during NF in those with and without antibiotic prophylaxis, although higher proportions were found in those NF events with prophylaxis when compared with those without (69.3% vs 59.4%, *p* = 0.18). Regarding antimicrobial (i.e., antibiotic and antifungal) prophylactic treatments by adverse medical complications of hypotension, impaired respiratory function, and abnormal chest X-ray in the first 3 and 5 days of NF, the analyses of between-group differences were not significant, showing that fewer medical complications were found in NF events in patients using prophylaxis than in those who were not. In total, since outcome data with deaths and pathogen isolates such as MRSA, *E. coli*, and others was limited, we did not conduct between-group comparison analyses on these data.

**Table 2:** Antimicrobial prophylaxis by microbiologically diagnosed infections, antibiotic modification during NF, gram-negative pathogens, gram-positive pathogens, and adverse medical complications during NF

	Antibiotic prophylaxis		
	Yes	No	
Outcome measures of fever	*n* (%)	*n* (%)	*p* values
MDBIs	47 (21.6)	15 (23.4)	0.88
Require modification of antibiotic during NF	151 (69.3)	38 (59.4)	0.18
Presence of gram-negative pathogens	29 (13.3)	7 (10.9)	0.78
Presence gram-positive pathogens	22 (10.1)	10 (15.6)	0.32
Hypotension in the first			
3 days of NF	26 (11.9)	10 (15.6)	0.57
5 days of NF	30 (13.8)	11 (17.2)	0.63
Impaired respiratory function in the first			
3 days of NF	25 (11.5)	8 (12.5)	0.10
5 days of NF	26 (11.9)	11 (17.2)	0.38
Abnormal chest X-ray in the first			
3 days of NF	32 (14.7)	8 (12.5)	0.81
5 days of NF	38 (17.4)	15 (23.4)	0.37
	Antifungal prophylaxis		
	Yes	No	
	*n* (%)	*n* (%)	
MDFIs	36 (18.7)	17 (19.1)	1.00
Hypotension in the first			
3 days of NF	24 (12.4)	12 (13.5)	0.96
5 days of NF	28 (14.5)	13 (14.6)	1.00
Impaired respiratory function in the first			
3 days of NF	21 (10.9)	12 (13.5)	0.67
5 days of NF	22 (11.4)	15 (16.9)	0.28
Abnormal chest X-ray in the first			
3 days of NF	26 (13.5)	14 (15.7)	0.75
5 days of NF	32 (16.6)	21 (23.6)	0.22

*p* Chi-square *p* value significant at < 0.05; *MDBIs* microbiologically diagnosed bacterial infections; *MDFIs* microbiologically diagnosed fungal infections

Table 3 shows the results of antibiotic and antifungal prophylaxis by total fever duration, CRP, and PCT. Similarly, there were no statistically significant differences between fever duration, CRP, and PCT between those using and those not using prophylaxis. Although all between-group differences showed no statistical significance, higher median fever duration, CRP, and PTC values were found in NF events with prophylaxis than in those without.

**Table 3:** Antimicrobial prophylaxis by NF duration, CRP, and PCT

	NF duration (hours)			
Treatments	Mean	SD	Median	*p* values
Antibiotics prophylactic				
No (*n* = 64)	183.9	185.7	109.5	0.95
Yes (*n* = 218)	178.9	176.3	124.5	
Antifungal prophylactic				
No (*n* = 89)	193.6	191.7	125	0.39
Yes (*n* = 193)	173.7	171.6	124	
	CRP > 5 (mg/L)*			
	Mean	SD	Median	
Antibiotics prophylactic				
No (*n* = 35)	78.9	72.4	53.0	0.26
Yes (*n* = 89)	76.9	49.7	69.0	
Antifungal prophylactic				
No (*n* = 44)	81.1	68.7	61.5	0.65
Yes (*n* = 80)	75.4	49.3	67.0	
	PCT ≥ 0.5 (ng/mL)**			
	Mean	SD	Median	
Antibiotics prophylactic				
No (*n* = 9)	37.0	105.4	0.88	0.34
Yes (*n* = 26)	6.5	11.3	1.34	
Antifungal prophylactic				
No (*n* = 13)	27.0	87.5	1.06	0.49
Yes (*n* = 22)	6.8	12.1	1.34	

*NF* neutropenic fever; *SD* standard deviation; *p* Mann–Whitney U test *p* value significant at < 0.05; *CRP* C-reactive protein; *PCT* procalcitonin; *abnormal values of CRP, when CRP normal reference value is ≤ 5 mg/L; **abnormal values of PCT, when PCT normal reference value is < 0.5 ng/mL

## Discussion

While considering leukemias as the target population under study, this research constitutes a considerable sample size of 282 post-chemotherapy NF events and adds to recent institutional findings with respect to previously scarce research on the effects of prophylactic antibiotic and antifungal treatments for post-chemotherapy NF in leukemias, primarily focused on the immediate outcome interests of NF. In terms of these outcome interests, CRP and PCT at the onset of fever, requiring antibiotic modification during NF, immediate adverse medical complications, the presence of gram-negative and gram-positive bacterial isolates, MDBIs, MDFIs, and the duration of NF, between-group analyses of those with and without prophylaxis analyses did not reveal significant differences. To some extent, the findings of this present institutional surveillance study might help to redefine the risk–benefit accounts of using antimicrobial prophylaxis, providing implications for service practice in the management of leukemias with chemotherapy.

In terms of the need for antibiotic modification after receiving first-line empirical antibiotic treatment, there were no significant differences between those who used and those who did not use prophylaxis. This might be interpreted as meaning that patients who underwent prophylactic treatments might not have exhibited a lesser pathogen load or experienced a significant benefit account from using prophylaxis in absolving second-line modified antibiotic treatments when compared with their counterparts who did not use prophylaxis. Investigators in previous research reported that patients who had received prophylactic antibiotics and developed NF had a significantly lower probability of response to the first-line empirical antibiotic [7]. Importantly and specifically, in our present study, NF events among those on antibiotic prophylaxis had higher CRP and PTC levels and a longer fever duration than in those who did not use prophylaxis, although between-group differences of these NF outcome measures were not significant. These group comparison findings might explain why patients who received prophylaxis had higher inflammatory biomarkers of CRP and PTC and might develop infections, requiring further modification of antibiotic treatments because of subsequent infections resistant to the first-line empirical antibiotic due to the use of/exposure to initial prophylaxis. Hence, patients might ultimately have fever for a longer time.

Our study findings, contrary to previous research [6, 7, 28], showed no significant between-group differences in microbiologically documented infections (MDBIs and MDFIs), inflammatory indicators of CRP and PTC, presence of gram-negative and gram-positive bacterial pathogens and the immediate medical complications of hypotension, impaired respiratory function, and abnormal chest X-ray findings. In addition, antifungal prophylactic treatment might reduce the diagnostic sensitivity of the galactomannan enzyme immunoassay for fungal infection [19], and such prophylaxis may pose possible subsequent challenges to the choice of strategy during ongoing management of post-chemotherapy neutropenia and NF; thus, researchers should remain cautious. Based on the above discussions, the benefit accounts of using antimicrobial prophylaxis appeared less supported.

The wider use of prophylactic antimicrobial treatments in the management of leukemias receiving chemotherapy has implications for service practice. Enhancing diagnostic laboratory surveillance on infection-related indicators and pathogen isolates and performing critical assessments of any adverse medical conditions (hypotension, impaired respiratory function, and chest infiltrations) during post-chemotherapy neutropenia are promising in guiding and delivering a precise prescription of prophylactic antimicrobial treatments, although to date, critical implementation of such practices has been limited. Instead of broad use of antimicrobial prophylaxis in leukemias with post-chemotherapy neutropenia, it is possible that critical diagnostic and medical complication surveillance and periodic evaluation of regional susceptibility and resistance patterns of pathogens during post-chemotherapy neutropenia and NF are realistic in redefining the risk–benefit accounts of using prophylaxis over time, taking together the considerations of the following premises. With recent advances in pharmacological technology, chemotherapeutic agents may be more potent, with less immunosuppression, and this may enhance patients’ immuno-protection against pathogens. In the era of microbial pandemics, ever-changing bacteriological patterns and the emergence of drug-resistant pathogens associated with the use of antimicrobial prophylaxis [8–11], health alertness, and self-awareness of healthcare precautions to minimize the risk of infection have been promoted at the individual level of the patient population. The toxicity of antimicrobial therapy and the emergence of antibiotic resistance associated with extensive use of prophylaxis have also been widely reported [13, 16]. Importantly, there were no significant differences or benefits in the immediate outcomes of post-chemotherapy NF between those who had undergone prophylactic treatments and those who had not, as reported in our present study. Majority of deaths had undergone antibiotic (13 deaths) and antifungal (10 deaths) prophylactic treatments. Specifically, prophylactic use of quinolones remains under discussion, primarily pertaining to a higher risk for gram-positive infection and the development of resistance, including toward MRSA [28]. In our institution, which is using quinolones as prophylactic treatments, we noted that pathogen isolates by MRSA (*n* = 17, from a total of 282 NF episodes) were higher when compared with data reported in a previous study (*n* = 5, from a total of 1358 bloodstream infections) [28]. Gram-positive bacterial isolates (*n* = 32, 11.3%) were slightly less predominant than gram-negative bacterial isolates (*n* = 36, 12.8%), in contrast to previously reported data in which gram-negative bacterial isolates were markedly predominant [29–31].

There were several limitations in our study, including that the risk for unmeasured or unidentifiable confounding factors could have resulted in over- and under-estimation of the effects of antimicrobial prophylaxis. We could not exclude the possibility that residual confounding, such as by baseline comorbid illness and first and non-first chemotherapy cycles, might have influenced our analysis. Small frequency counts of the outcome data among group comparison analyses should be cautioned, although the sample sizes of these data were comparable with previous studies [7, 16, 28]. There were fewer characteristics and outcome data, such as MRSA and deaths, which limited the ability to conduct between-group comparisons.

## Conclusion

Comparing group differences in terms of antimicrobial prophylaxis in post-chemotherapy NF in leukemias in our study did not show statistically significant differences in CRP and PCT at fever presentation, MDBIs, MDFIs, presence of gram-negative and gram-positive bacterial isolates, requiring modification of antibiotic treatments during NF, medical complications in the first 3 and 5 days of NF, or fever duration. Prophylactic antimicrobial treatment might not exhibit a lesser pathogen load and might result in subsequent infection that is resistant to the first-line empirical antibiotic due to the initial use of antibiotic prophylaxis during post-chemotherapy neutropenia. The benefit accounts of using prophylaxis are less supported. Use of antibiotic prophylaxis should be undertaken with caution due to a high risk of gram-positive infection and the development of resistance toward MRSA. Enhancing diagnostic laboratory surveillance and critical evaluation of adverse medical conditions during post-chemotherapy neutropenia and NF may provide insights to redefine the risk–benefit accounts of using antimicrobial prophylaxis.

## Data Availability

N/A

## Code availability

N/A
